# Aberrant expression of nuclear prothymosin α contributes to epithelial‐mesenchymal transition in lung cancer

**DOI:** 10.1002/1878-0261.70035

**Published:** 2025-04-21

**Authors:** Liyun Chen, Chung‐Teng Wang, Jia‐Ming Chang, Ai‐Li Shiau, Gia‐Shing Shieh, Yau‐Lin Tseng, Yi‐Ting Yen, Tang‐Hsiu Huang, Li‐Hsin Cheng, Yu‐Chih Wu, Chao‐Liang Wu, Bing‐Hua Su, Pensee Wu

**Affiliations:** ^1^ Department of Biochemistry and Molecular Biology, College of Medicine National Cheng Kung University Tainan Taiwan; ^2^ Department of Radiation Oncology Washington University School of Medicine St Louis MO USA; ^3^ Department of Microbiology and Immunology, College of Medicine National Cheng Kung University Tainan Taiwan; ^4^ Tong Yuan Diabetes Center, College of Medicine National Cheng Kung University Tainan Taiwan; ^5^ Thoracic Division, Department of Surgery Ditmanson Medical Foundation Chiayi Christian Hospital Taiwan; ^6^ Institute of Molecular Biology National Chung Cheng University Chiayi Taiwan; ^7^ Department of Urology, Tainan Hospital Ministry of Health and Welfare Taiwan; ^8^ Division of Thoracic Surgery, Department of Surgery, National Cheng Kung University Hospital, College of Medicine National Cheng Kung University Tainan Taiwan; ^9^ Division of Chest Medicine, Department of Internal Medicine, National Cheng Kung University Hospital, College of Medicine National Cheng Kung University Tainan Taiwan; ^10^ Core Laboratory of Organoids Technology Office of R&D, Taipei Medical University Taiwan; ^11^ School of Respiratory Therapy, College of Medicine Taipei Medical University Taiwan; ^12^ TMU Research Center of Thoracic Medicine Taipei Medical University Taiwan; ^13^ Keele Medical School Keele University UK

**Keywords:** epithelial–mesenchymal transition, lung cancer, prothymosin α, Smad7 acetylation, TGF‐β

## Abstract

Elevated expression of prothymosin α (ProT) is frequently observed in cancers, but the underlying molecular mechanism remains poorly understood. Here, we report the clinical relevance of ProT expression and its correlation with lung cancer progression. We have shown that ProT was highly expressed in early‐stage lung cancer, exhibiting nuclear localization; on the contrary, a loss of nuclear ProT expression was detected in late‐stage tumor specimens. Furthermore, the expression of nuclear ProT impaired lung cancer cell migration, suppressed TGF‐β‐induced epithelial‐to‐mesenchymal transition (EMT)‐associated transcription factor expression, and inhibited *in vivo* tumor metastasis. The suppressive effect of ProT was further found to trigger Smad7 acetylation‐dependent deregulation of TGF‐β signaling. ProT enhanced Smad7 stability by promoting its lysine acetylation, thereby competing with the binding of Smad2 to the *SNAI1*, *TWIST1*, and *ZEB1* promoters. Eventually, the binding of Smad7 in the presence of ProT resulted in reduced expression of the EMT transcription factors, leading to the inhibition of TGF‐β‐induced EMT and tumor metastasis. Collectively, this study unravels the role of ProT in lung cancer progression and highlights the potential of nuclear ProT as an indicator for monitoring tumor development.

## Graphical abstract

Nuclear prothymosin α inhibits epithelial‐mesenchymal transition (EMT) in lung cancer by increasing Smad7 acetylation and competing with Smad2 for binding to *SNAI1*, *TWIST1*, and *ZEB1* promoters. In early‐stage cancer, ProT suppresses TGF‐β‐induced EMT, while its loss in the nucleus in late‐stage cancer leads to enhanced EMT and poor prognosis.

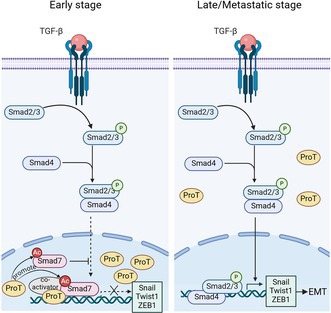

**Abbreviations**
CBP: CREB‐binding protein
ChIP: chromatin immunoprecipitation
DMFS: distant metastasis‐free survival
EMT: epithelial‐to‐mesenchymal transition
FBS: fetal bovine serum
GAPDH: glyceraldehyde 3‐phosphate dehydrogenase
GFP: green fluorescent protein
IHC: immunohistochemistry
IP: immunoprecipitation
Luc: luciferase
MMPs: matrix metalloproteinases
NLS: nuclear localization signal
OS: overall survival
PBS: phosphate‐buffered saline
PFS: progression‐free survival
ProT: prothymosin α
qPCR: quantitative real‐time polymerase chain reaction
SEM: standard error of the mean
shRNA: short‐hairpin RNA
Smad7: mothers against decapentaplegic homolog 7
Snail: Zinc finger protein SNAI1
TGF‐β1: transforming growth factor‐β
TIMPs: inhibitors of metalloproteinases
Twist1: twist‐related protein 1
ZEB1: zinc finger E‐box‐binding homeobox 1

## 1 Introduction

Prothymosin α (ProT), a 12.5‐kDa acidic protein encoded by the *PTMA* gene, is highly conserved and widely distributed in a variety of tissues [1, 2]. Elevated expression of ProT in various cancers, which may serve as a potential prognostic biomarker, has been investigated in several studies. For instance, high expression of ProT at either mRNA or protein levels has been observed in hepatocellular carcinoma [3], neuroblastoma [4], as well as head and neck, breast, and colorectal cancers [5, 6, 7]. In lung cancer, expression of ProT is also correlated with poor prognosis [8]. Our previous cohort study of 149 lung cancer patients has indicated that high levels of ProT expression are more likely to be observed in smokers, squamous cell carcinoma, and patients aged under 65 years, which are associated with a high recurrence rate [9]. However, most of these studies have used immunostaining to detect ProT expression, while the underlying molecular mechanism of ProT in cancer progression remains largely unknown.

Given its nuclear localization signals mapped at residues 87–88 and 101–104 [2], human ProT is considered a nuclear protein, and its intra‐nuclear expression is indeed immunohistochemically observed in most human tissues [10] and clinical tumor specimens [7, 11]. Despite its predominant localization in the nucleus, increasing evidence has suggested the cytoplasmic and extracellular localization of ProT under certain pathological conditions [12, 13]. We have shown that cytoplasmic ProT is a more significant prognostic factor for tumor recurrence than nuclear ProT in upper urinary tract cancer [14]. Furthermore, not only is the elevated expression of ProT detected in bladder cancer patients, but the lack of nuclear ProT expression is also associated with Foxp3‐positive lymphocyte infiltration and shorter progression‐free survival [15]. Translocation of ProT from the nucleus to the cytoplasm has been suggested to be associated with cell apoptosis, where the nuclear localization signal of ProT is disrupted by caspase cleavage [16]. ProT can be detectable at extracellular levels, although the secretory signal and mechanisms of ProT have yet to be determined. Notably, extracellular ProT has long been reported to possess immunomodulatory activities [17].

In our previous studies, we have discovered that homozygous ProT transgenic mice spontaneously develop enlarged airspace and destruction of alveolar tissue displaying pathological phenotypes of emphysema [18]. Moreover, overexpression of ProT causes disruption of the transforming growth factor‐β (TGF‐β1) signaling, leading to the imbalance between matrix metalloproteinases (MMPs) and tissue inhibitors of metalloproteinases (TIMPs), thereby further contributing to emphysema development [19]. Both studies have demonstrated the critical role of ProT in pulmonary physiology.

Here, we examined the expression of ProT in lung cancer patients and found that changes in ProT expression levels correspond to disease progression. More importantly, the location of ProT shifted from the nucleus to the cytoplasm in patients with metastatic lung cancer, suggesting the potential involvement of ProT in promoting tumor metastasis. Given the findings that ProT can interrupt TGF‐β signaling, which is the fundamental pathway for initiating epithelial‐to‐mesenchymal transition (EMT), and the loss of nuclear ProT in lung cancer progression, we aimed to elucidate the molecular mechanism and association between ProT and lung cancer metastasis.

## 2 Materials and methods

### 2.1 Human specimens

Clinical specimens were collected from lung cancer patients who underwent surgery at the Thoracic Division, Department of Surgery, National Cheng Kung University (NCKU) Hospital, Tainan, Taiwan. The timeframe during which lung cancer specimens were collected was from 1st Aug 2017 to 21st Feb 2024. The study methodologies conformed to the standards set by the Declaration of Helsinki. Prior to participation, all subjects were informed of the details of the study and provided written consent. Informed consent was obtained from all subjects, and the experimental protocol was approved by the Human Experiment and Ethics Committee NCKU Hospital. All clinical samples were obtained with the approval of the Institutional Review Board of NCKU Hospital (NCKUH, IRB number: B‐ER‐105‐406).

### 2.2 Cell culture and treatments

Human A549 lung cancer cell lines (RRID: CVCL_0023) were obtained from the Bioresource Collection and Research Center (BCRC, Taiwan). Human H1299 lung cancer cell lines (RRID: CVCL_0060) were obtained from the American Type Culture Collection (ATCC). Human 293T (Lenti‐X™ 293T, RRID: CVCL_4401) embryonic kidney cells were obtained from Takara Bio USA (San Jose, CA, USA), Inc. All cell lines have been authenticated in the past 3 years, and all experiments were performed with mycoplasma‐free cells (Certificate of Analysis for A549 cells, Catalog No. Lot Number 60074, BCRC; Certificate of Analysis for 293T cells, Catalog No. Lot Number 632180, Takara). The H1299 cells have been genotyped and matched to the ATCC STR database (hsSTR‐0905Y24). Cells were cultured in Dulbecco's Modified Eagle Medium (DMEM) supplemented with 10% fetal bovine serum (FBS) and 50 μg·mL^−1^ gentamicin at 37 °C. Where indicated, cells were treated with 10 ng·mL^−1^ of recombinant TGF‐β1 (PeproTech, Thermo Fisher Scientific Inc., Waltham, MA, USA) in DMEM with 2% FBS for the indicated time.

### 2.3 Lentiviral vectors and plasmids

Lentiviral vectors pWPXL‐ProT‐IRES‐GFP encoding ProT and GFP, pWPT encoding GFP, pLKO.1‐Flag‐Smad7‐HA encoding Smad7, and pLKO.1‐Flag‐Smad7(K64R/K70R)‐HA encoding Smad7 (K64R/K70R) with lysine residues 64 and 70 replaced with arginine have been described previously [18, 19]. For knockdown experiments, pLKO.1‐puro‐based lentiviral vectors expressing short‐hairpin RNA (shRNA) specific for human ProT (TRCN0000135421) and luciferase (Luc) (TRCN0000072246) were obtained from the National RNAi Core Facility, Academia Sinica, Taiwan. Various recombinant lentiviruses expressing ProT, GFP, Smad7, Smad7 (K64R/K70R), ProT shRNA, and Luc shRNA were produced by transient transfection of 293T cells with the aforementioned lentiviral vectors along with the packaging plasmid psPAX2 and the VSV‐G expression plasmid pMD2G as previously described [20]. Plasmids pcDNA3.1‐ProT and pcDNA3.1 have been described previously [19].

### 2.4 Migration and invasion assays

A549 cells were pretreated with TGF‐β1 for 24 h and seeded for the migration assay. For Transwell assays, cells were placed onto the upper chamber of the Transwell filter with 8 μm pores (Thermo Fisher Scientific, Waltham, MA, USA), and the bottom well contained regular growth media. After incubation for 6 h, migrated cells were fixed with methanol and stained with 0.1% Giemsa. Migration was quantified by cell counts in three random fields (×100 magnification) per sample. Data represent three independent experiments in each group, performed in triplicate. For wound healing assays, confluent cells were wounded using a 200‐μL pipette tip and cultured in DMEM with 2% FBS in the presence of mitomycin C (5 μg·mL^−1^). After incubation for 8 h, cells were visualized under a light microscope. Cell migration was calculated by measuring the final wound area compared to the initial area. To conduct the invasion assay, A549 cells were seeded on Transwell inserts (BD Biosciences, Franklin Lakes, NJ, USA) with a thin layer of collagen type I (3 mg·mL^−1^, BD Biosciences) in the presence of 10% FBS and allowed to invade across the collagen for 24 h. Subsequently, the cells that had invaded through the insert membrane were fixed, stained with SYTOX Green (Invitrogen, Thermo Fisher Scientific Inc., Waltham, MA, USA), and counted using a fluorescence microscope.

### 2.5 RNA extraction and quantitative real‐time PCR (qPCR)

Total RNA was isolated using TRIzol Reagent (Invitrogen) according to the manufacturer's instruction, and 2 μg RNA was used for cDNA synthesis performed with High Capacity cDNA Reverse Transcription kit (Applied Biosystems, Thermo Fisher Scientific Inc., Waltham, MA, USA). Gene expression analysis was evaluated by qPCR using QuantiNova SYBR green PCR kit (Qiagen) in triplicates and performed on Roter‐Gene Q (Qiagen, Qiagen, Hilden, Germany) according to manufacturer's recommendations. The relative expression levels of mRNA were normalized to *GAPDH* and the fold change was calculated using the $2^{-\Delta \Delta C_{t}}$ method. The following primers were used for qPCR: human *SNAI1* (5′‐TCGGAAGCCTAACTACAGCGA‐3′ and 5′‐AGATGAGCATGGCAGCGAC‐3′); human *TWIST1* (5′‐GTCCGCAGTCTTACGAGGGAG‐3′ and 5′‐TGGAGGACCTGGTAGAGGAA‐3′); human *ZEB1* (5′‐CAGCTTGATACCTGTGAAGGG‐3′ and 5′‐TATCTTGTGGTCGTGTGGGACT‐3′); human *SMAD7* (5′‐CCCCATCACCTTAGCCGACTCTGC‐3′ and 5′‐CCCAGGGGCCAGATAATT‐3′); human *GAPDH* (5′‐ACTTCAACAGCACACCCACT‐3′ and 5′‐GCCAAATTCGTTGTCATACCAG‐3′).

### 2.6 Immunoblotting and immunoprecipitation

Cells were homogenized in RIPA lysis buffer containing protease inhibitor cocktail, and proteins were quantified using a BCA assay (Thermo Fisher Scientific) according to the manufacturer's instructions. Total proteins (20–40 μg) were separated by 10–12% SDS/PAGE, transferred onto PVDF membranes, and blocked with 5% semi‐skimmed milk. Membranes were incubated overnight at 4 °C with primary antibodies against E‐cadherin (BD), N‐cadherin (BD), Snail (Cell Signaling, Danvers, MA, USA), Twist1 (Sigma‐Aldrich, Burlington, MA, USA), ZEB1 (Novus, Bio‐Techne, Minneapolis, MI, USA), acetylated lysine (Cell Signaling, Danvers, MA, USA), Flag epitope (Sigma), ProT (clone 2F11; ascites fluid), and β‐actin (Abcam, Cambridge, UK). Anti‐Flag M2 gel (Sigma) was used for immunoprecipitation to detect the Flag‐conjugated protein. Horseradish peroxidase (HRP)‐conjugated goat anti‐mouse IgG and goat anti‐rabbit IgG (Cell Signaling) were used as secondary antibodies where appropriate, and protein‐antibody complexes were visualized by the ECL system (MilliporeSigma, Burlington, MA, USA) and the Biospectrum AC imaging system (UVP).

### 2.7 Chromatin immunoprecipitation (ChIP)

Cells were transduced with lentiviral vectors encoding different proteins and treated with or without TGF‐β1. ChIP was performed as previously described [18]. Briefly, proteins were cross‐linked with 1% formaldehyde and washed with cold PBS, and cells were lysed with SDS lysis buffer. The cell lysate was sonicated to shear DNA to lengths between 500 and 1000 bp, followed by immunoprecipitation with anti‐Flag‐M2 affinity gel (Sigma‐Aldrich) or with protein A plus G sepharose (Santa Cruz) combined with anti‐Smad2 (Genetex, Irvine, CA, USA) or anti‐IgG antibodies (Santa Cruz, Biotechnology, Dallas, TX, USA). The immunoprecipitates were washed, eluted for the protein/DNA complexes, and reverse cross‐linked. PCR was performed on the DNA that was purified using the phenol‐chloroform‐extracted method to detect the binding sites for Smad7 and Smad2 within *SNAI1*, *TWIST1*, and *ZEB1* promoters with specific primers. The PCR primers used in ChIP included *SNAI1* (5′‐CGCTCCGTAAACACTGGATAA‐3′ and 5′‐GAAGCGAGGAAAGGGACAC‐3′), *TWIST1* (5′‐ACCTTCCGAGGCGTAGTCCTT‐3′ and 5′‐GGAGTTCCAAAGGCCAAACC‐3′), and *ZEB1* (5′‐TCATCATCAAGGGAACTCCC‐3′ and 5′‐AGGTAAAGTTGGAGGCTCGG‐3′) primers. The PCR products were separated by 1% agarose gel electrophoresis.

### 2.8 Histology and immunostaining

For histology, formalin‐fixed paraffin‐embedded human and mouse lung tissue sections were processed with hematoxylin and eosin (H&E) staining after deparaffinization and rehydration. For immunohistochemistry, tissue sections were blocked with BSA after deparaffinization and rehydration, followed by incubation with primary antibodies at 4 °C overnight. Primary antibodies included monoclonal antibodies against ProT (clone 2F11, ascites fluid) [21], Smad7 (R&D, Bio‐Techne, Minneapolis, MI, USA), Snail (Abcam), Twist1 (Genetex), and ZEB1 (Novus). Subsequently, HRP‐conjugated goat anti‐mouse or anti‐rabbit IgG (Jackson Laboratory, Bar Harbor, ME, USA) antibodies were added and incubated at room temperature for 2 h. The reactivity was visualized with aminoethyl carbazole (red color, Zymed, Thermo Fisher Scientific Inc., Waltham, MA, USA) and counterstained with hematoxylin. Secondary antibodies used for immunofluorescence staining were Alexa‐fluor 594 goat anti‐mouse IgG (Invitrogen), and nuclei were stained with DAPI (Sigma) under a dark room‐temperature‐humidified environment. Images were captured using a constant exposure time by an Olympus microscope. Photographs from immunohistochemistry were digitally processed to obtain the integrated optical density (IOD), and data were analyzed using the metamorph software (Molecular Devices, San Jose, CA, USA).

### 2.9 Mouse tumor models

A549 cells were transduced with lentiviral vectors encoding ProT and GFP to establish A549/ProT and A549/GFP cells overexpressing ProT and GFP, respectively. Groups of 8‐week‐old male NOD.Cg‐Prkdcscid/JNarl (NOD/SCID) mice (approximately 22 g) were subcutaneously inoculated with 4 × 10^6^ A549/ProT or A549/GFP cells. Palpable tumors were measured in two perpendicular axes every 3 to 4 days with a Vernier caliper, and tumor volumes were calculated as (length of tumor) × (width of tumor)^2^ × 0.45. Mice were sacrificed when tumor volumes exceeded 2000 mm^3^. Tumors and lungs were collected at the time of euthanasia. Mice were purchased from the Laboratory Animal Center of National Cheng Kung University (NCKU), Tainan, Taiwan. All mice were housed in groups of three to four per cage within an air‐conditioned vivarium, with free access to food and water. Throughout the study, a 12‐h light/dark cycle was maintained, with lights being activated at 8 am. The animal experimental protocol complied with the Animal Protection Act of Taiwan and gained approval from the Institutional Animal Care and Use Committee of NCKU (IACUC approval no. 98023).

### 2.10 Statistical analysis

Statistical analyses were performed using the graphpad prism software (Graphpad Software Inc., La Jolla, CA, USA). All data were presented as mean ± standard error of the mean (SEM) from at least three independent replicates for each experiment. Statistical significance was determined by Student's *t*‐test or one‐way ANOVA. The overall survival (OS), progression‐free survival (PFS), and distant metastasis‐free survival (DMFS) curves were calculated by the Kaplan–Meier survival method and compared by the log‐rank test. Correlations were measured using Pearson's correlation coefficient (r). Any *P‐*value of < 0.05 is regarded as statistically significant.

## 3 Results

### 3.1 Expression of nuclear ProT is reduced in advanced lung tumor specimens

**Fig. 1:**
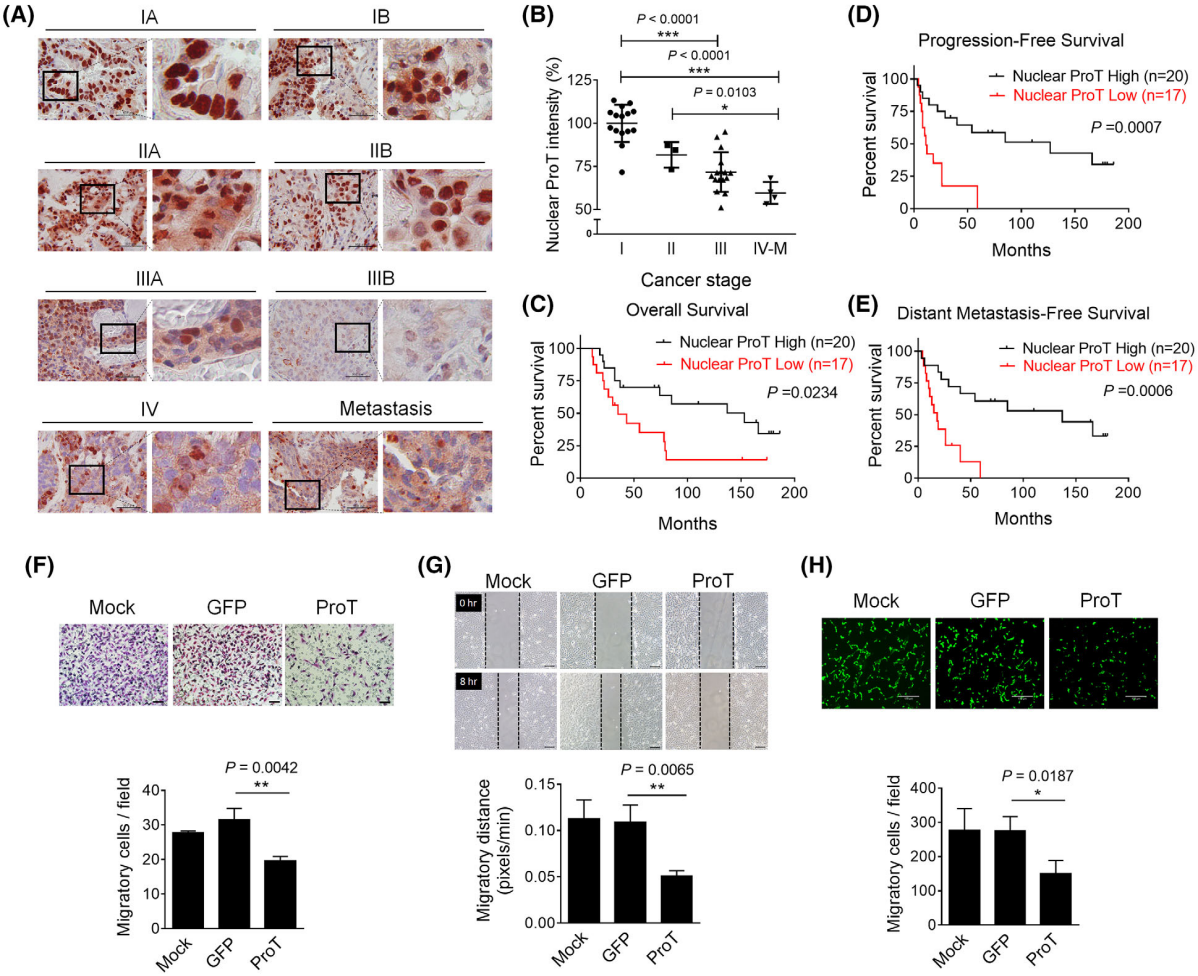
Decreased expression of nuclear ProT in advanced lung tumor specimens and suppression of migration and invasion of lung cancer cells by ProT. (A) Immunohistochemical detection of ProT in clinical lung tumor sections with various tumor stages. Scale bars shown on 400× images correspond to 50 μm, and the boxed areas are magnified right on each panel. (B) Quantification of nuclear ProT immunointensity ProT was analyzed by metamorph software. Values shown are levels of immunointensity in individual specimens, with the mean level in stage I tumors arbitrarily set to 100, in three randomly selected fields in each section (*n* = 15 for stage I; *n* = 3 for stage II; *n* = 16 for stage III; *n* = 4 for stage IV‐M). (C–E) Low levels of nuclear ProT expression were correlated with poor prognosis in patients with lung cancer. Immunointensity for nuclear ProT was individually categorized as low or high according to the mean value of total specimens (*n* = 20 for nuclear ProT high, *n* = 17 for nuclear ProT low). Kaplan–Meier curves of OS (C), PFS (D), and DMFS (E) in patients with high or low nuclear ProT expression. Differences in survival intervals were analyzed by the log‐rank test and *P*‐values less than 0.05 were shown in the charts of Kaplan–Meier curves. (F) Migration of A549 (Mock) (*n* = 3), A549/GFP (*n* = 3), and A549/ProT (*n* = 3) cells following treatment with TGF‐β1 (10 ng·mL^−1^) for 24 h, as assessed by the Transwell assay. The migratory cells were stained and quantified by counting three fields (at 100 × magnification) in each section. Scale bars shown on 200× images correspond to 50 μm. (G) Migratory capabilities of A549 (*n* = 3), A549/GFP (*n* = 3), and A549/ProT (*n* = 3) cells determined by the wound healing assay. Wound closure of scrape‐wounded cell monolayers was examined after 8 h by light microscopy, photographed, and quantified by measurement of the wound area. Scale bars shown on 200× images correspond to 50 μm. The vertical lines indicate the wound formed at 0 h (top panel) and 8 h (bottom panel). (H) Invasive capabilities of A549 (*n* = 3), A549/GFP (*n* = 3), and A549/ProT (*n* = 3) cells following treatment with TGF‐β1 (10 ng·mL^−1^) for 24 h were determined using Transwell inserts pre‐coated with collagen type 1. The invasive cells were stained and quantified by counting three fields (at 100× magnification) in each section by fluorescence microscopy. Scale bars shown on 200× images correspond to 170 μm. The *P*‐values of (C–E) were calculated using the log‐rank test. The quantification values and error bars of (B) and (F–H) shown were mean ± SEM, and *P*‐values less than 0.05 were shown in the quantitative charts (*P < 0.05, **P < 0.01, ***P < 0.001, two‐tailed unpaired *t*‐test). DMFS, distant metastasis‐free survival; GFP, green fluorescent protein; OS, overall survival; PFS, progression‐free survival; ProT, prothymosin α; SEM, standard error of the mean; TGF‐β1, transforming growth factor‐β.

Expression of ProT was immunohistochemically detected in a cohort of 38 lung tumor specimens from patients at different stages, among which four patients with stage IV lung cancer developed distant metastasis (stage IV‐M). As shown in Fig. 1A, expression levels of ProT in early‐stage lung cancer (stage I, *n* = 15; stage II, *n* = 3) were higher than those in later‐stage lung cancer (stage III, *n* = 16; stage IV, *n* = 4). However, quantification of ProT immunointensity reveals that increased expression of ProT was not significantly associated with lung cancer progression (Fig. S1). Notably, ProT was predominantly localized in the nucleus in early‐stage tumors. In contrast, nuclear expression of ProT was markedly reduced in later‐stage tumors (Fig. S1). Quantification of nuclear ProT immunointensity shows that nuclear ProT expression was inversely correlated with lung cancer progression (Fig. 1B). Notably, metastatic lung tumors (stage IV‐M) had the lowest nuclear expression of ProT and tended to lose nuclear expression. We further evaluated the correlation of nuclear ProT levels with patient survivals in the same cohort (Fig. 1A) of lung cancer patients. One patient in our cohort was excluded for survival analysis because of death during the surgical procedure. The immunointensity for nuclear ProT was individually categorized as low or high (Fig. 1B) according to the mean value (*n* = 20 for nuclear ProT high, *n* = 17 for nuclear ProT low). Kaplan–Meier survival analysis reveals that patients with nuclear ProT low‐expressing tumors had significantly shorter survivals, including overall survival (OS) (Fig. 1C), progression‐free survival (PFS) (Fig. 1D), and distant metastasis‐free survival (DMFS) (Fig. 1E) than those with nuclear ProT high‐expressing tumors (Log‐rank test, *P* = 0.0234 for OS; *P* = 0.0007 for PFS; *P* = 0.0006 for DMFS). Collectively, these results suggest that nuclear ProT expression is reduced in lung cancer progression and correlated with lung cancer prognosis.

### 3.2 ProT suppresses TGF‐β1‐induced migration and expression of EMT‐associated transcription factors

Given high levels of nuclear ProT expression in early‐stage lung tumors and loss of nuclear ProT in metastatic tumors, we next investigated the function of nuclear ProT in relation to cancer cell metastasis. We generated ProT‐overexpressing and vector control A549 human lung cancer cells using LV‐ProT/LV‐GFP‐mediated gene transfer. In A549/ProT cells, nuclear expression of ProT was detected, whereas cytoplasmic ProT was only slightly detectable (Fig. S2A). A549/ProT, A549/GFP, and parental cells were examined for their migratory capabilities after treatment with TGF‐β for 24 h by Transwell and wound healing assays. In the Transwell assay, the number of migratory cells was significantly reduced in A549/ProT cells compared with that in A549/GFP or parental cells (Fig. 1F). Similarly, in the wound healing assay, A549/ProT cells displayed delayed gap closure compared with their two control counterparts (Fig. 1G). In the invasion assay using Transwell inserts pre‐coated with collagen type 1, a significant reduction in the number of invasive cells was noted in A549/ProT cells as compared to that observed in A549/GFP or parental cells (Fig. 1H). Taken together, these results indicate that overexpression of ProT suppresses the migration and invasion of lung cancer cells in the presence of TGF‐β.

**Fig. 2:**
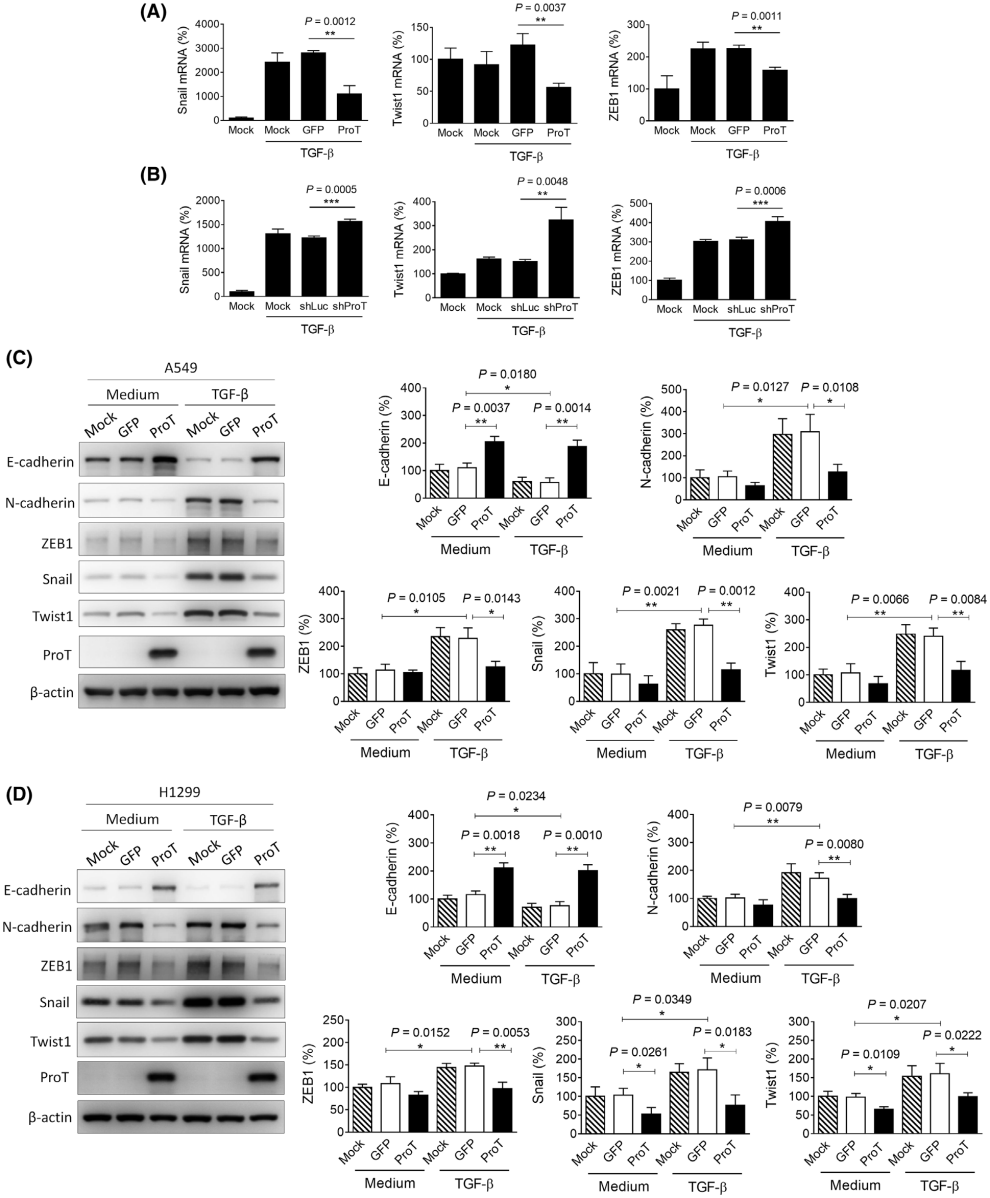
ProT inhibits TGF‐β‐induced EMT by downregulating the expression of EMT‐associated transcription factors. (A and B) Detection of Snail, Twist1, and ZEB1 mRNA expression by RT‐qPCR in A549 (*n* = 3), A549/GFP (*n* = 3), and A549/ProT (*n* = 3) cells (A), as well as in A549 cells transduced with lentiviral vectors expressing shRNA specific to ProT (shProT) (*n* = 3) or luciferase (shLuc) (*n* = 3) (B) following treatment with TGF‐β (10 ng·mL^−1^) for 24 h. Relative expression levels are normalized to *GAPDH*, and ratios of control cells were arbitrarily set to 100. (C, D) Detection and quantification of EMT‐associated markers in mock (*n* = 3)‐, GFP (*n* = 3)‐, and ProT (*n* = 3)‐transduced A549 (C) and H1299 (D) cells in the presence or absence of TGF‐β1 (10 ng·mL^−1^) for 48 h by immunoblotting. Expression of β‐actin served as the loading control. Representative immunoblots from three independent experiments and quantitative analysis of the indicated proteins are shown. Ratios between the intensity of the bands corresponding to the indicated protein and those corresponding to β‐actin were calculated, and ratios of control cells were arbitrarily set to 100. All quantification values and error bars shown were mean ± SEM, and *P*‐values less than 0.05 were shown in the quantitative charts (*P < 0.05, **P < 0.01, ***P < 0.001, two‐tailed unpaired *t*‐test). GFP, green fluorescent protein; Luc, luciferase; ProT, prothymosin α; SEM, standard error of the mean; shRNA, short‐hairpin RNA; Snail, Zinc finger protein SNAI1; TGF‐β1, transforming growth factor‐β; Twist1, Twist‐related protein 1; ZEB1, Zinc finger E‐box‐binding homeobox 1.

TGF‐β is known to induce expression of EMT‐associated transcription factors and markers, such as Snail, Twist, Zeb, and N‐cadherin, and downregulate the cell–cell adhesion molecule E‐cadherin, resulting in enhanced cell migration [22]. Significant elevation of mRNA levels of Snail and ZEB1, but not Twist1, was observed in A549 cells upon TGF‐β treatment (Fig. 2A). Notably, overexpression of ProT significantly suppressed not only Snail and ZEB1, but also Twist1 mRNA expression in A549 cells following TGF‐β treatment (Fig. 2A). Accordingly, knockdown of endogenous ProT expression enhanced TGF‐β‐induced Snail, Twist1, and ZEB1 mRNA expression in A549 cells (Fig. 2B). Immunoblot analysis also shows that TGF‐β‐induced upregulation of EMT‐associated molecules, including N‐cadherin, Snail, Twist1, and ZEB1, was abrogated by ProT overexpression in A549 (Fig. 2C) and H1299 (Fig. 2D) human lung cancer cells. However, ProT had no effects on TGF‐β‐induced downregulation of E‐cadherin (Fig. 2C,D). Taken together, overexpression of ProT attenuates the impact of TGF‐β‐induced EMT in A549 and H1299 lung cancer cells, suggesting a regulatory role for ProT in TGF‐β signaling in lung cancer.

### 3.3 ProT interrupts TGF‐β signaling via regulation of Smad7 stabilization

**Fig. 3:**
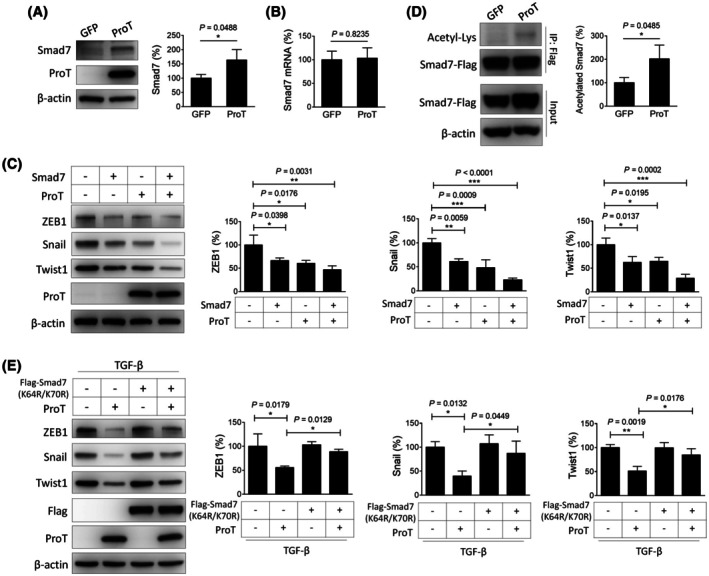
Prot suppresses Snail, Twist1, and ZEB1 expression via promoting Smad7 acetylation. (A) Detection and quantification of Smad7 in A549/GFP (*n* = 3) and A549/ProT (*n* = 3) cells by immunoblotting. (B) Detection of Smad7 mRNA expression in A549/GFP (*n* = 4) and A549/ProT (*n* = 4) cells by RT‐qPCR. Relative expression levels are normalized to *GAPDH*, and ratios of control cells were arbitrarily set to 100. (C) Detection and quantification of ZEB1, Snail, and Twist1 by immunoblotting in H1299 cells transiently transfected with pcDNA3.1‐ProT (*n* = 3), pLKO.1‐Flag‐Smad7 (*n* = 3), or a control vector (pcDNA3.1) (*n* = 3) for 24 h. Overexpression of ProT was confirmed in cells transfected with pcDNA3.1‐ProT by immunoblotting. (D) Detection of acetylated Smad7 in A549/GFP (*n* = 3) and A549/ProT (*n* = 3) cells transduced with Flag‐Smad7 and immunoprecipitated with anti‐Flag antibody, followed by immunoblotting for acetyl‐lysine. (E) Detection and quantification of ZEB1, Snail, and Twist1 by immunoblotting in A549/GFP (*n* = 3) and A549/ProT (*n* = 3) cells transduced with Flag‐Smad7(K64R/K70R), which contains lysine to arginine mutations at residues 64 and 70, following treatment with TGF‐β1 for 24 h. Overexpression of ProT and Smad7(K64R/K70R) was confirmed in cells transduced with ProT and/or mutant Smad7 by immunoblotting with antibodies against ProT or Flag. (A, C, D, E). Expression of β‐Actin serves as the loading control. Representative immunoblots from three independent experiments and quantitative analysis of the indicated proteins are shown. Ratios between the intensity of the bands corresponding to the indicated proteins and those corresponding to β‐Actin were calculated, and ratios of control cells were arbitrarily set to 100. The quantification values and error bars displayed were mean ± SEM, and *P*‐values less than 0.05 were indicated in the quantitative charts. *P < 0.05, **P < 0.01, ***P < 0.001, one‐way ANOVA (C) or two‐tailed unpaired *t*‐test (A, D, E). Acetyl‐Lys, acetylated lysine; GFP, green fluorescent protein; IP, immunoprecipitation; ProT, prothymosin α; SEM, standard error of the mean; Smad7, mothers against decapentaplegic homolog 7; Snail, Zinc finger protein SNAI1; TGF‐β1, transforming growth factor‐β; Twist1, Twist‐related protein 1; ZEB1, Zinc finger E‐box‐binding homeobox 1.

The TGF‐β pathway transduces its signaling through activating Smad proteins, while the inhibitory Smad Smad7 binds to type I TGF‐β receptor and blocks the recruitment of Smad2 and Smad3, leading to negative regulation of TGF‐β signaling [23]. In A549/ProT cells, the protein level of Smad7 was elevated (Fig. 3A), but its mRNA level was similar (Fig. 3B), as compared with that of vector control A549/GFP cells, suggesting that ProT modulates Smad7 expression through post‐translational regulation.

Snail is primarily an essential inducer of EMT [24]. As Snail was the most ProT‐downregulated gene among the three EMT‐associated transcription factors we studied (Fig. 2A), we next used H1299 cells, which endogenously express Snail (Fig. S2B), to investigate whether overexpression of ProT inhibited Snail expression via regulating Smad7 protein. We examined the expression of Snail in H1299 cells after transfection with ProT or Smad7 expression vectors by immunoblot analysis. Figure 3C shows that levels of Snail were reduced in cells transfected with either ProT or Smad7 expression vector. Notably, cotransfection with both ProT and Smad7 expression vectors further decreased its levels. In addition, similar results were shown for the levels of Twist1 and ZEB1 (Fig. 3C).

ProT can bind histone to interact with several transcription factors and transcriptional coactivators [25, 26], including CREB‐binding protein (CBP)/p300 that is involved in Smad7 acetylation against ubiquitin‐mediated degradation [27, 28]. To confirm that ProT increased Smad7 acetylation, A549/ProT and A549/GFP cells, which were further transduced with lentiviral vectors encoding Flag‐tagged Smad7, were analyzed by immunoprecipitation with anti‐Flag antibody, followed by immunoblotting with antibodies against acetylated lysine or Smad7. Figure 3D shows that overexpression of ProT in A549 cells increased Smad7 acetylation by direct interaction between Smad7 and ProT (Fig. 3D). We then used Flag‐tagged Smad7(K64R/K70R), in which lysine residues 64 and 70 known to be acetylated by p300 were both mutated to arginine [19], to confirm that the inhibition of TGF‐β‐induced Snail expression by ProT indeed involved enhanced Smad7 acetylation. Overexpression of ProT inhibited TGF‐β‐induced Snail expression, whereas this suppressive effect was not observed in A549 cells cotransduced with ProT and mutant Smad7(K64R/K70R) (Fig. 3E).

### 3.4 ProT regulates Smad7‐mediated transcriptional repression of the *SNAI1* , *TWIST1* , and *ZEB1* genes by enhancing Smad7 acetylation and sequestering it in the nucleus

**Fig. 4:**
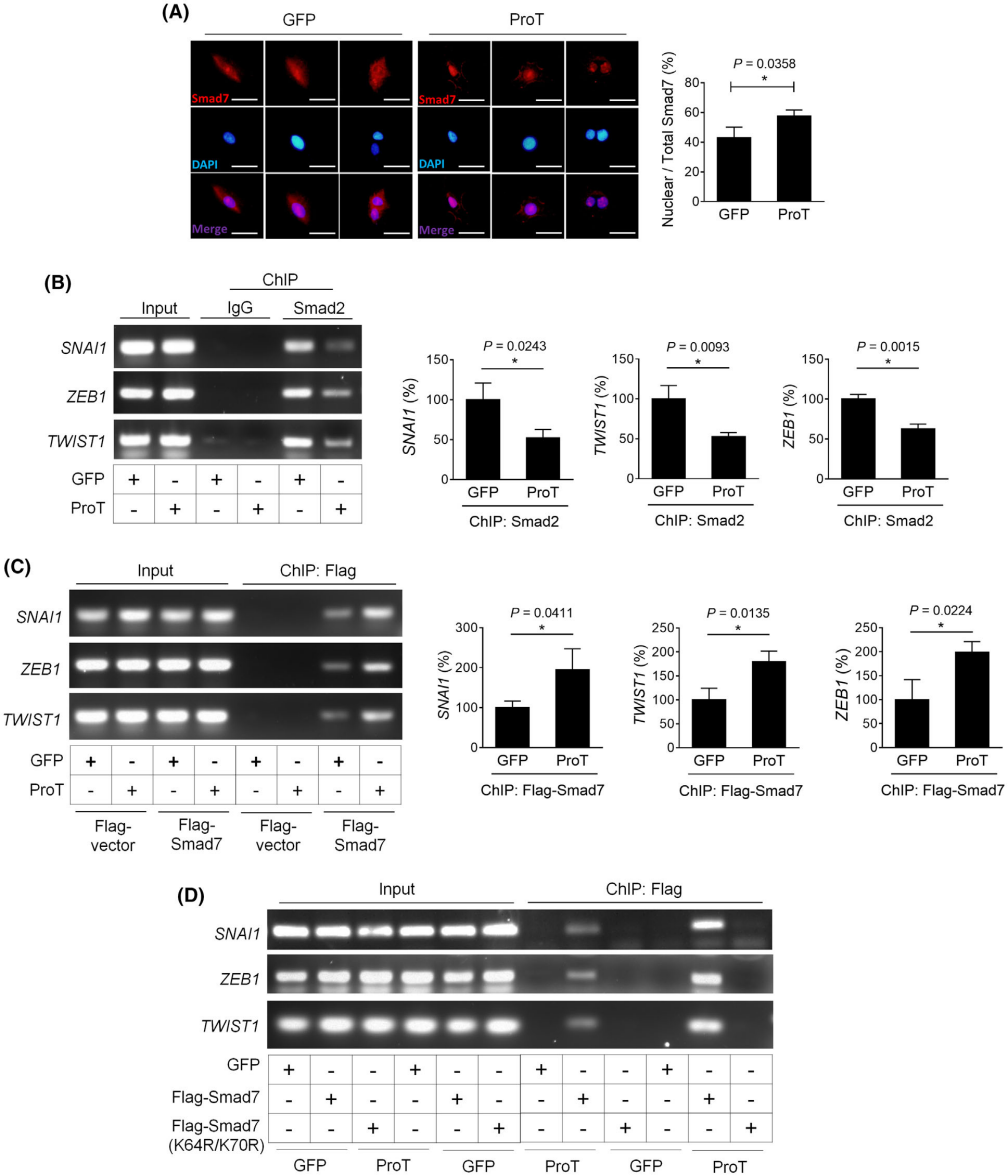
ProT promotes Smad7 nuclear localization and enhances Smad7‐mediated transcriptional repression of *SNAI1*, *TWIST1*, and *ZEB1* genes. (A) Immunofluorescence staining of A549/GFP (*n* = 3) and A549/ProT (*n* = 3) cells with mouse anti‐human Smad7 antibody, followed by Alexa‐fluor 594‐conjugated goat anti‐mouse IgG after treatment with TGF‐β1 (10 ng·mL^−1^) for 3 h. Nuclei were counterstained with DAPI. Fluorescence signals for Smad7 (red) and the nucleus (blue) were examined by fluorescence microscopy. The merged column represents the superposition of the cells stained with anti‐Smad7 and DAPI. Scale bars shown on 400× images correspond to 20 μm. Quantification of fluorescence intensity of nuclear Smad7 was analyzed by the imagej software (National Institutes of Health, Bethesda, MD, USA), and values shown are nuclear : total Smad7 fluorescence ratios. (B) ChIP analysis of Smad2 binding to *SNAI1*, *TWIST1*, and *ZEB1* promoters in A549/GFP (*n* = 3) and A549/ProT (*n* = 3) cells following treatment with TGF‐β1 for 3 h. (C) ChIP analysis of Smad7 binding to *SNAI1*, *TWIST1*, and *ZEB1* promoters in A549/GFP (*n* = 3) and A549/ProT (*n* = 3) cells transduced with Flag‐Smad7 following treatment with TGF‐β1 for 3 h. (B, C) Representative PCR results from three independent experiments and quantitative analysis of the indicated genes are shown, and ratios of control cells were arbitrarily set to 100. The quantification values and error bars of (A–C) displayed were mean ± SEM, and *P*‐values less than 0.05 were indicated in the quantitative charts (*P < 0.05, two‐tailed unpaired *t*‐test). (D) ChIP analysis of Smad7, but not Smad7(K64R/K70R), binding to *SNAI1*, *TWIST1*, and *ZEB1* promoters. 293T cells that had been transduced with lentiviral vectors encoding ProT (*n* = 3) or GFP (*n* = 3) were further transduced with Flag‐Smad7. Immunoprecipitation was done using anti‐Flag M2 magnetic beads. ChIP, chromatin immunoprecipitation; GFP, green fluorescent protein; ProT, prothymosin α; SEM, standard error of the mean; Smad, mothers against decapentaplegic homolog; Snail, Zinc finger protein SNAI1; Twist1, Twist‐related protein 1; ZEB1, Zinc finger E‐box‐binding homeobox 1.

As ProT was mainly identified in the nucleus of non‐metastatic lung tumors, we sought to determine whether ProT regulated the inhibitory function of Smad7 in the nucleus. A549 cells that had been transduced with ProT or GFP were treated with TGF‐β1 and the localization of Smad7 was examined by immunofluorescence staining. In A549/GFP cells, Smad7 was localized in both the cytoplasm and nucleus (Fig. 4A). Interestingly, Smad7 was mainly observed in the nucleus in most A549/ProT cells (Fig. 4A), suggesting that Smad7 may be sequestered by ProT within the nucleus, thereby exerting its repressive effects on the *SNAI1* gene in the nucleus.

Promoters of human *SNAI1*, *TWIST1*, and *ZEB1* genes contain Smad‐binding element (SBE) normally recognized by Smad2/Smad3 to induce transcriptional activities [24, 29]. ChIP assay reveals that Smad2 binding to *SNAI1*, *TWIST1*, and *ZEB1* promoters was reduced in A549/ProT cells compared with that in A549/GFP cells after TGF‐β treatment (Fig. 4B). Moreover, in A549 cells that were transduced with ProT or GFP alone, or further transduced with Flag‐Smad7, Smad7 was found to bind to *SNAI1*, *TWIST1*, and *ZEB1* promoters in cells transduced with ProT and Flag‐Smad7, as evidenced by the ChIP assay (Fig. 4C). In the ChIP analysis of Smad7 binding to *SNAI1*, *TWIST1*, and *ZEB1* promoters, the binding was detected in 293T cells transduced with wild‐type Flag‐Smad7, but not Smad7(K64R/K70R) mutant, indicating that Smad7 acetylation is required for the binding (Fig. 4D). Notably, overexpression of ProT increased Smad7 binding to *SNAI1*, *TWIST1*, and *ZEB1* promoters (Fig. 4D). Therefore, combining our previous data [19] with those found here, we conclude that ProT enhances nuclear Smad7 acetylation and thereby competes with the binding of Smad2 on *SNAI1*, *TWIST1*, and *ZEB1* promoters, resulting in repressing Snail, Twist1, and ZEB1 expression.

### 3.5 Human lung cancer cells with nuclear ProT expression are associated with low metastatic potential *in vivo*

**Fig. 5:**
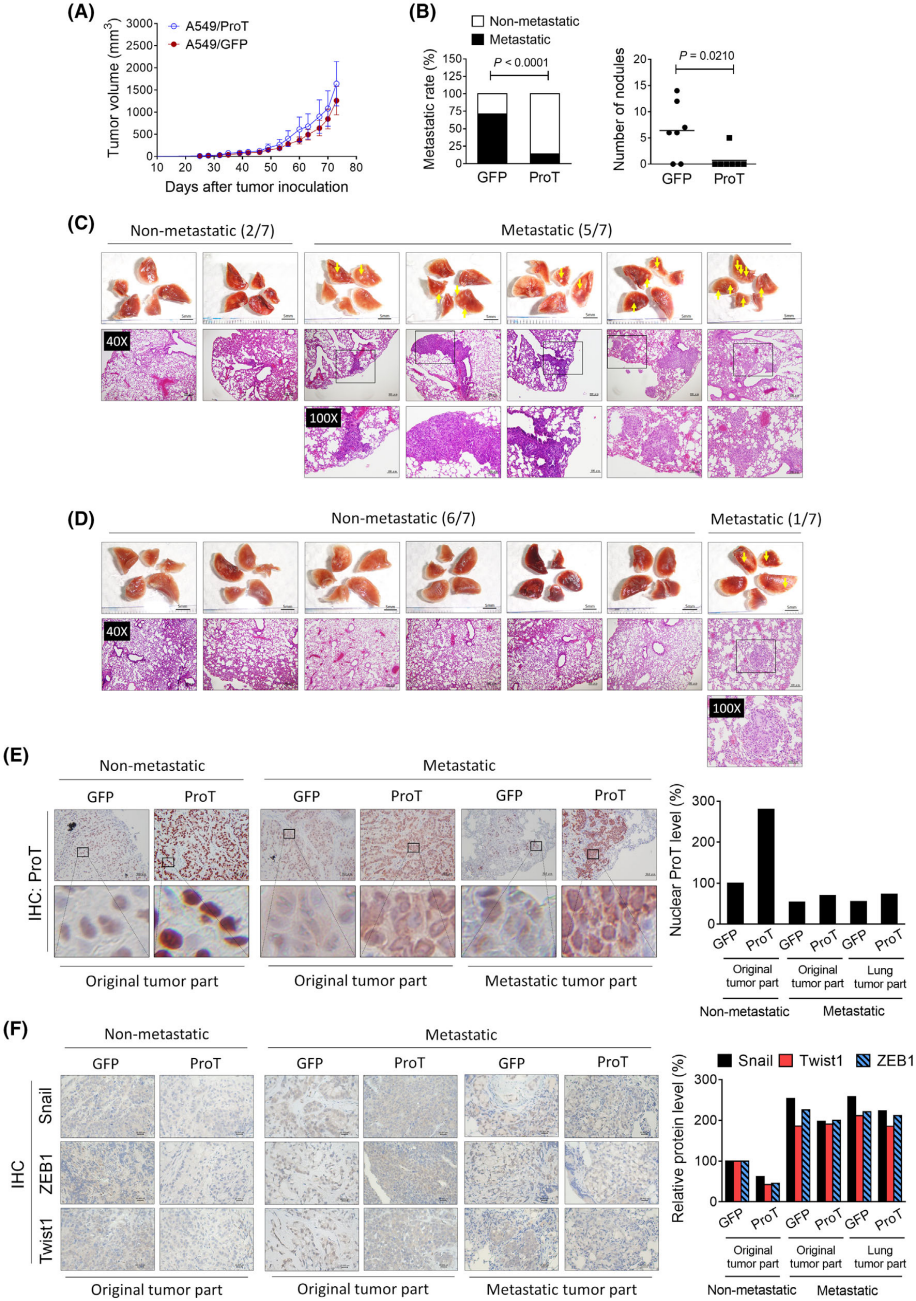
Nuclear ProT‐expressing lung tumors exhibit low metastatic potential *in vivo* and express low levels of Snail. (A) Tumor volumes in mice bearing A549/ProT or A549/GFP tumors. NOD/SCID mice were subcutaneously inoculated with A549/ProT (*n* = 7) or A549/GFP (*n* = 7) cells (4 × 10^6^) at day 0, and tumor volumes were measured every 3–4 days. Mice were sacrificed when their tumor volumes exceeded 2000 mm^3^. Error bars indicated mean ± SEM. (B) The contingency of lung metastatic tumor was measured using Fisher's exact test (*left*, *P* < 0.0001). The number of metastatic nodules was counted and assessed using the Mann–Whitney *U*‐test (*right*, *P* = 0.0210). (C and D) Gross appearance (upper panels) and histology (lower panels) of the lungs from A549/GFP (C) and A549/ProT (D) tumor‐bearing mice. Arrowheads indicate tumor nodules. Scale bars shown on 1×, 40×, and 100× images correspond to 5 mm, 200 μm, and 100 μm. (E) Immunohistochemical detection (*left*) and quantification (*right*) of ProT in non‐metastatic and metastatic (original and lung tumor parts) tumor tissues. Scale bars shown on 100× images correspond to 50 μm, and the boxed areas are magnified right on each panel. (F) Immunohistochemical detection (*left*) and quantification (*right*) of Snail, Twist1, and ZEB1 in non‐metastatic and metastatic (original and lung tumor parts) tumor tissues. Scale bars shown on 200× images correspond to 20 μm. The *P*‐values of (B) were calculated using the Fisher's exact test or Mann–Whitney *U*‐test. GFP, green fluorescent protein; IHC, immunohistochemistry; ProT, prothymosin α; SEM, standard error of the mean; Snail, Zinc finger protein SNAI1; Twist1, Twist‐related protein 1; ZEB1, Zinc finger E‐box‐binding homeobox 1.

To further evaluate the impact of ProT on tumor growth and metastasis *in vivo*, we generated A549/ProT and A549/GFP cells that stably overexpress ProT and GFP, respectively, and subcutaneously inoculated tumor cells into the right flank of NOD/SCID mice. All mice were monitored for tumor growth after tumor inoculation, and tumor volumes were measured. There were no significant differences in tumor growth between the mice inoculated with A549/ProT cells and those inoculated with A549/GFP cells (Fig. 5A). Gross appearances of metastatic lung nodules produced by A549/GFP and A549/ProT cells were compared. Metastatic nodules were observed and calculated in the lungs of five out of seven A549/GFP tumor‐bearing mice, which exhibited severe lung metastasis evidenced by H&E staining (Fig. 5B,C). In marked contrast, small lung metastatic nodules were detected only in one of seven A549/ProT tumor‐bearing mice, while the remaining six mice had no observable lung metastasis (Fig. 5D). In addition, the expression of ProT in the tumors from A549/ProT tumor‐bearing mice with or without lung metastasis was examined. Intense nuclear staining of ProT was observed in non‐metastatic tumors, whereas ProT staining was less intense, and there was an absence of nuclear staining in metastatic tumors (Fig. 5E). Furthermore, the expression of Snail, Twist1, and ZEB1 was higher in metastatic tumors than in non‐metastatic tumors (Fig. 5F). It appears that tumors with higher intensity of nuclear ProT staining displayed lower intensity of Snail, Twist1, and ZEB1 staining and were more likely to be non‐metastatic. Moreover, Snail, Twist1, and ZEB1 staining show increased intensity in metastatic tumors, which may contribute to lung metastasis in mice bearing subcutaneous tumors.

The nuclear localization signal (NLS) at the C‐terminus of ProT is required for its nuclear import and transcriptional activity. We next investigated whether cytoplasmic ProT had direct effects on promoting tumor metastasis. In addition to A549/ProT and A549/GFP cells, we generated stable A549/ProTΔNLS cells that express ProT lacking the NLS sequence in the cytoplasm. We compared tumor growth and metastasis in NOD‐SCID mice that had been subcutaneously inoculated with A549/ProTΔNLS, A549/ProT, or A549/GFP. Mice inoculated with A549/ProTΔNLS cells exhibited delayed tumor growth compared to those inoculated with A549/ProT or A549/GFP cells (Fig. S3A). Surprisingly, although most A549/ProTΔNLS tumors were under 1000 mm^3^ in size after 81 days of tumor cell inoculation, four out of seven mice had lung tumor nodules, indicating tumor metastasis (Fig. S3B). It is of particular interest that despite the tumor sizes being smaller than others, lung metastasis occurred.

### 3.6 Low expression of Smad7 and high expression of Snail, Twist1, and ZEB1 correlated with poor prognosis in lung cancer patients

**Fig. 6:**
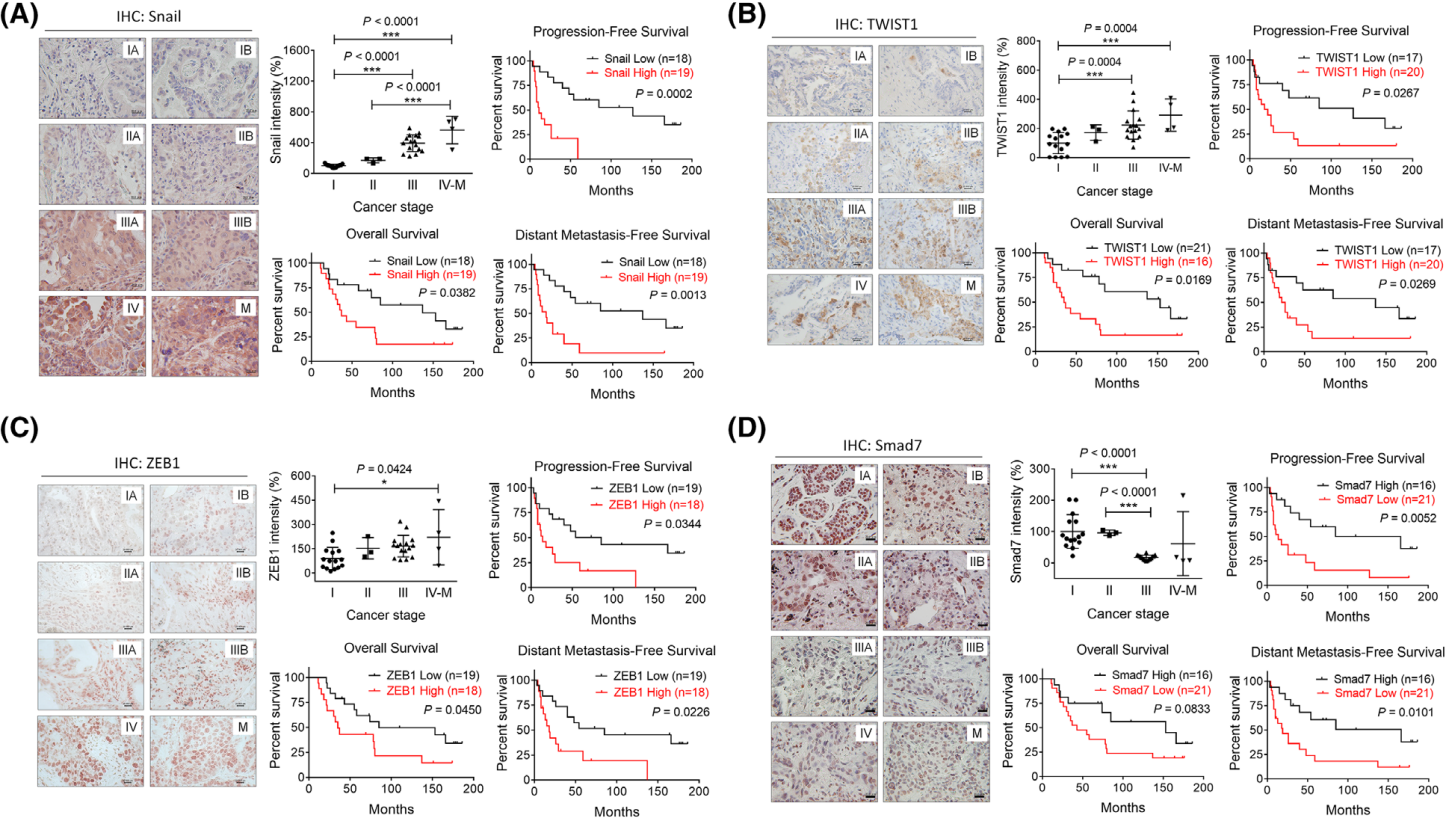
Expression of Snail, Twist1, ZEB1, and Smad7 proteins and their correlations with patient survivals in lung cancer. (A–D) Immunohistochemical staining for Snail (A), Twist1 (B), ZEB1 (C), and Smad7 (D) in tumor specimens from 38 lung cancer patients, including 15 stage I, 3 stage II, 16 stage III, and 4 stage IV/metastasis (M) samples. Scale bars shown on 400× images correspond to 20 μm. Levels of immunointensity of Snail, Twist1, ZEB1, and Smad7 were analyzed by three randomly selected fields within the same specimen and quantified. Values shown are the relative immunointensity, with the level in stage I tumors arbitrarily set to 100. Quantification values and error bars of immunohistochemical staining displayed were mean ± SEM, and *P*‐values less than 0.05 were indicated in the quantitative charts (*P < 0.05, ***P < 0.001, two‐tailed unpaired *t*‐test). Immunointensity of the indicated proteins was categorized as low and high in tumor specimens of patients with lung cancer. Kaplan–Meier curves of OS, PFS, and DMFS in patients with high or low expression of Snail (A), Twist1 (B), ZEB1 (C), and Smad7 (D). Differences in survival intervals were analyzed by the log‐rank test, and *P*‐values less than 0.05 were shown in the charts of Kaplan–Meier curves. DMFS, distant metastasis‐free survival; IHC, immunohistochemistry; M, metastasis; OS, overall survival; PFS, progression‐free survival; ProT, prothymosin α; SEM, standard error of the mean; Smad7, mothers against decapentaplegic homolog 7.

We initially identified the association of decreased nuclear ProT expression with lung cancer progression, mainly seen in metastatic lung tumors (Fig. 1A–E). The protein expression of Snail, Twist1, ZEB1, and Smad7 in these tumors was further evaluated by immunohistochemical staining. Expression of Snail (Fig. 6A), Twist1 (Fig. 6B), and ZEB1 (Fig. 6C) gradually increased from stage I to stage IV tumors with high expression levels at metastasis‐stage tumors. Furthermore, there were statistically significant differences in OS, PFS, and DMFS between patients with low and high expression of Snail (Fig. 6A), Twist1 (Fig. 6B), and ZEB1 (Fig. 6C), indicating that increased expression of Snail, Twist1, and ZEB1 was correlated with lung cancer progression and poor patient survival. Although the expression of Smad7 varied in stage I tumors, it decreased in stages II, III, and IV‐M tumors. A significant statistical reduction of Smad7 expression was not observed, which may be due to the low sample numbers and inevitable variation in metastatic specimens (Fig. 6D). Nevertheless, significant discrepancies were observed in PFS and DMFS between patients exhibiting low and high Smad7 expression (Fig. 6D), suggesting a correlation between decreased Smad7 expression and lung cancer progression and poor patient survival.

### 3.7 Aberrant nuclear ProT expression is associated with an increased EMT process and poor prognosis in lung cancer

**Fig. 7:**
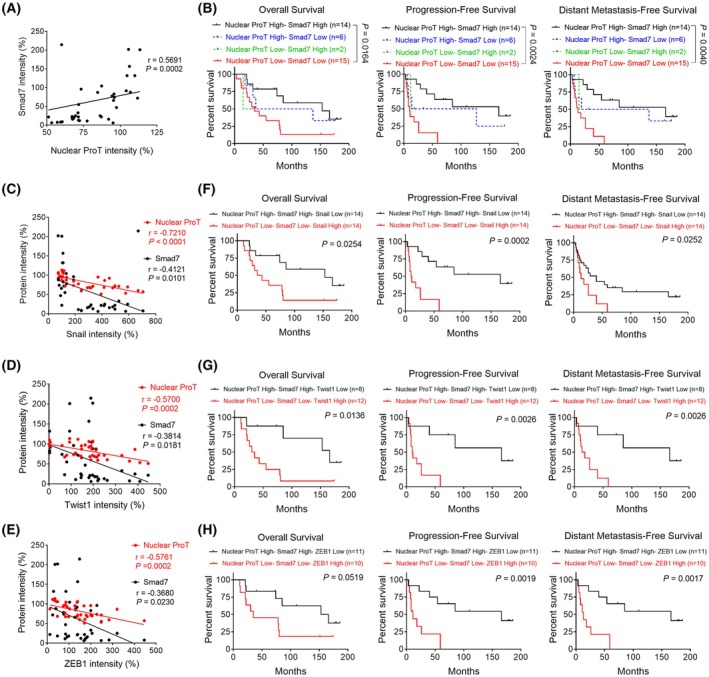
Low expression of nuclear ProT is associated with the EMT process and poor prognosis in lung cancer. (A) A positive correlation (*P* < 0.05; Pearson's correlation coefficient) between levels of nuclear ProT immunointensity and those of Smad7 immunointensity. (B) Kaplan–Meier curves of OS, PFS, and DMFS in patients with high or low expression of nuclear ProT and Smad7. (C–E) Negative correlations (*P* < 0.05; Pearson's correlation coefficient) between levels of Snail (C), Twist1 (D), or ZEB1 (E) immunointensity and those of nuclear ProT or Smad7. (F–H) Kaplan–Meier curves of OS, PFS, and DMFS in patients with high or low expression of Snail (F), Twist1 (G), or ZEB1 (H), as well as nuclear ProT and Smad7. The *P*‐values of (A, C, E, G) were analyzed by Pearson's correlation coefficient. Differences in survival intervals were analyzed by the log‐rank test and *P*‐values less than 0.05 were shown in the charts of Kaplan–Meier curves (B, F, D, H). DMFS, distant metastasis‐free survival; OS, overall survival; PFS, progression‐free survival; ProT, prothymosin α; Smad7, mothers against decapentaplegic homolog 7.

**Fig. 8:**
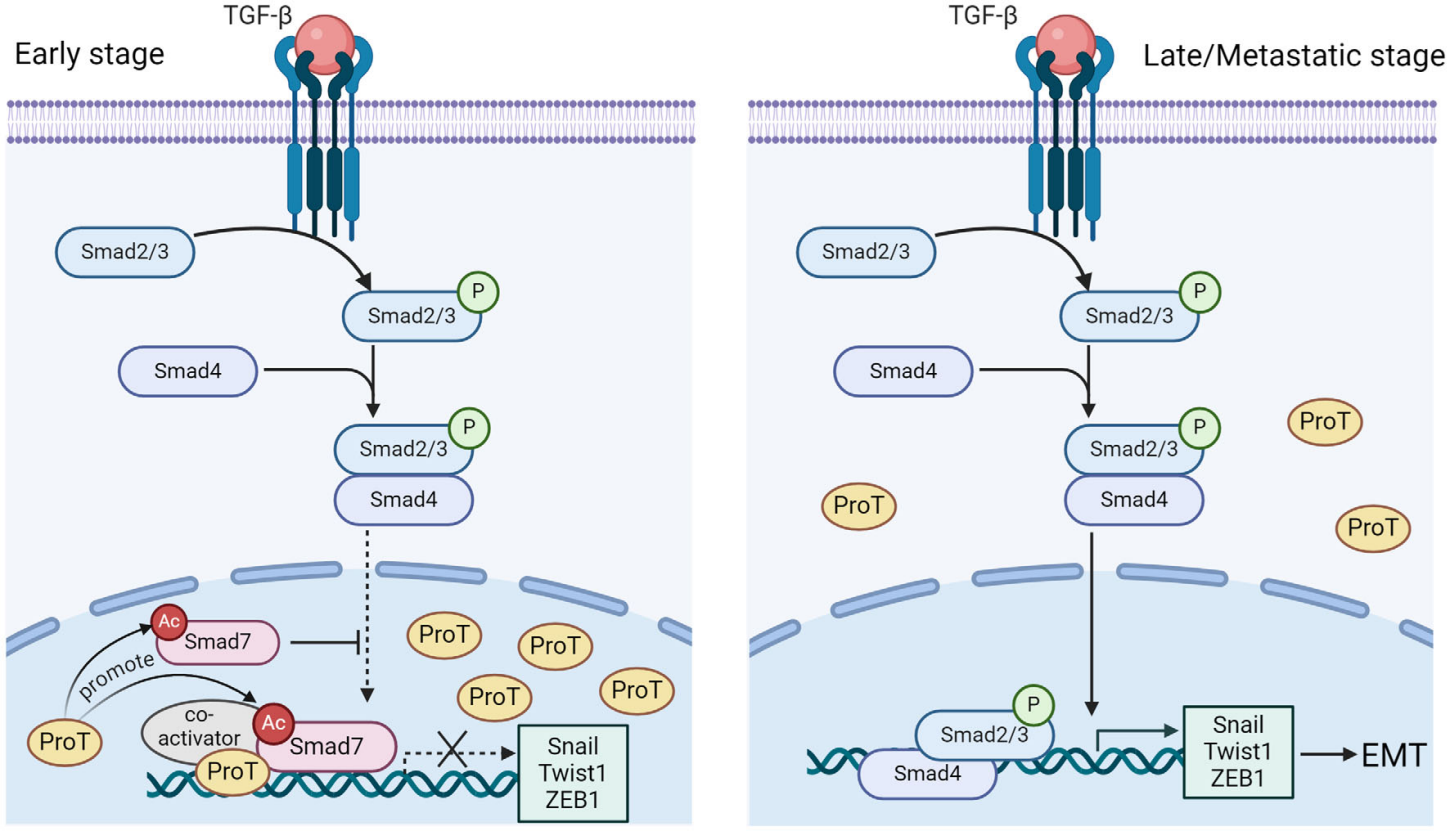
Proposed model for the inhibition of EMT by nuclear ProT through enhancing Smad7 acetylation and competing with the binding element of Smad2/3 on *SNAI1*, *TWIST1*, and *ZEB1* promoters in lung cancer. Nuclear ProT expression decreases with lung cancer progression and correlates with poor prognosis. In the early stage of lung cancer, overexpression of nuclear ProT attenuates TGF‐β‐induced EMT, suggesting a regulatory role for ProT in TGF‐β signaling. ProT increases Smad7 acetylation, leading to Smad7‐mediated transcriptional repression of *SNAI1*, *TWIST1*, and *ZEB1*. By increasing Smad7 binding to EMT gene promoters, ProT competes with Smad2 to repress Snail, Twist1, and ZEB1 expression. In the late/metastatic stage of lung cancer, the decrease of nuclear ProT loses its role in repressing EMT. The aberrant loss of nuclear ProT correlates with heightened EMT and poor clinical outcomes in lung cancer patients.

Next, we investigated the roles of aberrant nuclear ProT expression and decreased Smad7 in EMT‐related lung cancer progression. Figure 7A shows that there is a positive correlation between the expression levels of ProT and Smad7 in lung cancer. When the two determinants were analyzed in combination, patients with low nuclear ProT and low Smad7 expression exhibited a worse outcome with regard to OS, PFS, and DMFS compared to those with high nuclear ProT and high Smad7 expression (Fig. 7B). Furthermore, levels of Snail (Fig. 7C), Twist1 (Fig. 7D), and ZEB1 (Fig. 7E) expression were negatively correlated with levels of nuclear ProT as well as with Smad7. The combination of low Smad7 and low nuclear ProT expression with high Snail, Twist1, and ZEB1 expression was correlated with a worse outcome for OS, PFS, and DMFS compared with the combination of high Smad7 and high nuclear ProT expression with low Snail (Fig. 7F), Twist1 (Fig. 7G), and ZEB1 (Fig. 7H) expression. Consequently, high expression of Snail, Twist1, and ZEB1 in conjunction with low expression of Smad7 and nuclear ProT was correlated with a poor prognosis in patients with lung cancer. Collectively, our results demonstrate that the expression of nuclear ProT is associated with the suppression of EMT via enhancing Smad7 acetylation, which impacts lung cancer progression (Fig. 8).

## 4 Discussion

In the present study, we explored the regulatory mechanism of nuclear ProT in relation to tumor metastasis. The expression of nuclear ProT in lung cancer cells acts to slow or prevent EMT by abrogating the TGF‐β signaling, which is highly activated in lung cancer progression [30]. ProT can stabilize Smad7 by enhancing the acetylation of its lysine residues, interrupting the expression of Snail, a TGF‐β‐induced EMT‐associated transcription factor. The mechanism was further identified to regulate the promoter binding activity of Smad7 that competes with the binding of Smad2 to the *SNAI1* promoter, resulting in reduced transcriptional activity of Snail.

ProT is highly expressed in many types of cancers [15, 31, 32]. Indeed, we also identified elevated levels of ProT expression in early‐stage lung tumors, confirming an early involvement of ProT in the process of tumor formation. The transcriptional activity of ProT was reported to be regulated by the transcription factor E2F and the c‐myc oncogene, which are directly associated with several mitogenic pathways [33, 34, 35]. Besides, increased expression with cyclin proteins between S and G2/M phases of the cell cycle [34, 36] and the interaction with STAT3 nuclear translocation [37] are most likely to indicate the regulatory role of ProT in promoting tumor development. In addition, we analyzed a single‐cell RNA sequencing dataset of 22 patients with lung adenocarcinoma (GSE148071). Patients were divided into two groups on the basis of their distribution of ProT transcript levels, also known as *PTMA*: *PTMA*‐Low (*PTMA*/L, *n* = 11) and *PTMA*‐High (*PTMA*/H, *n* = 11) (Fig. S4A). *PTMA* is highly expressed in epithelial cells and also in stromal and immune cells. Tumors with high *PTMA* gene expression in epithelial cells correlated with high *SMAD7* gene expression compared to those in the *PTMA*‐L group (Fig. S4B, red dotted line). Gene set enrichment analysis shows that epithelial cells in the *PTMA*‐H group had significantly higher enrichment scores for TGF‐β signaling and EMT pathways versus the *PTMA*‐L group, indicating that elevated expression of *PTMA* is associated with increased tumor metastatic potential (Fig. S4C). Moreover, analysis of clinical data from The Cancer Genome Atlas (TCGA) lung adenocarcinoma (LUAD) cohort (*n* = 502) revealed that *PTMA* expression levels are significantly elevated in lung cancer stages I–IV compared to normal tissue specimens. However, no significant differences in *PTMA* expression were observed between different cancer stages (I–IV). There was no significant difference in overall survival rates between patients with high *PTMA* expression and those with low *PTMA* expression (Fig. S5). Although elevated *PTMA* expression was related to tumor growth and proliferation, we found that significantly high levels of nuclear ProT protein expression decreased in late‐stage lung tumor specimens. We discovered that ProT hinders the migration of lung cancer cells *in vitro* and reduces the incidence of tumor metastasis *in vivo*. These results may seem contradictory to high ProT expression in lung cancer and other cancers. However, it is worth noting that thymosin α1, a peptide fragment derived from ProT, can slow down urethane‐induced lung tumorigenesis in mice and increase the survival of lung tumor‐bearing mice [38, 39]. Although ProT is frequently associated with poor prognosis, our results clearly indicate that nuclear ProT suppresses the EMT process, thereby acting as a negative regulator in lung cancer metastasis. Furthermore, ProT is widely expressed in normal tissues [40, 41], and homozygous and heterozygous ProT knockout mice either die at an early age or show impaired neurogenic differentiation [42], suggesting an essential role for ProT in tissue development. Thus, it is inconclusive whether ProT is an oncoprotein. Evidence has indicated that ProT is involved in cell division and proliferation by modulating the chromatin remodeling process [36, 43] and anti‐apoptosis by inhibiting apoptosome formation and caspase‐3 activation [44], as well as possessing extracellular neuroprotective effects [45, 46, 47]. Undoubtedly, ProT is a multifunctional molecule; however, its biological role needs to be further characterized.

The nuclear localization signal (NLS) at the C‐terminus of ProT is indispensable for its nuclear import. Aberrant nuclear ProT and high levels of cytoplasmic ProT may facilitate lung cancer poor prognosis and cell metastasis (Fig. 1A–E and Fig. S3). A549/ProTΔNLS cells, which overexpressed ProT lacking NLS, exhibited delayed tumor growth in tumor‐bearing mice (Fig. S3A). Despite the small tumor size of the primary tumor, lung metastasis still occurred (Fig. S3B). The nuclear form of ProT contains a motif TKKQK at the C terminus, which is a potent NLS [48, 49]. The cytoplasmic form of ProT lacks the NLS, which is cleaved primarily at D99 by activated caspase‐3 and may relocate to the cytoplasm or cell membrane [44, 45, 50]. Caspase‐3, a cysteine protease with established functions in apoptosis, has been identified as a crucial factor in the progression of lung cancer beyond its traditional role [51, 52]. Its frequent expression in non‐small cell lung cancer is associated with a poor prognosis, indicating caspase‐3 as a potential prognostic marker in lung cancer [53, 54]. Nevertheless, the pathological role of caspase‐3 in lung cancer remains largely unexplored. In the current study, we postulated that caspase‐3 may cleave the NLS of ProT, leading to its relocation from the nucleus to the cytoplasm and consequently impeding the regulatory role of ProT in mediating transcriptional repression of EMT in lung cancer via Smad7. Further investigation is required to elucidate the precise mechanisms underlying the intracellular translocation of ProT and to ascertain whether cytoplasmic ProT gains functions to regulate EMT in lung cancer.

The inhibitory role of Smad7 is mainly characterized by its regulatory function in the cytoplasm, including preventing Smad2/3 phosphorylation and interrupting Smad2/3 and Smad4 complex formation and their nuclear accumulation [55]. The nuclear role of Smad7 is less understood and varies in different biological settings. For instance, nuclear Smad7 functioning via a TGF‐β‐independent mechanism acts as a nuclear coactivator essential for the myogenic differentiation of myoblasts [56]. At the same time, some studies have indicated a nuclear corepressor role of Smad7 with respect to its interaction with histone proteins and transcription factors, such as SIRT1, HDAC1, and E2F [57, 58, 59]. In the present study, nuclear Smad7 was also found to serve as a transcriptional suppressor of Snail gene expression through ProT‐regulated nuclear distribution and stabilization. In the presence of ProT, high levels of Smad7 were detected by immunoblotting and nuclear localization by immunostaining. Nevertheless, increased expression of Smad7 was not observed at the transcriptional levels. These results prompted us further to investigate the regulatory relationship between nuclear Smad7 and ProT. Knowing that histone acetyltransferase p300 acetylates Smad7 on lysines 64 and 70, thereby preventing the ubiquitination of these lysine residues and leading to its stabilization [28], we generated a mutant Smad7 that lacked acetylated lysines and demonstrated that ProT failed to exert its suppressive effect on Snail expression via mutant Smad7. In fact, ProT has been shown to enhance protein acetylation in various diseases, including NF‐κB in emphysema [18], STAT3 in polycystic kidney disease [37], and P53 in cancer cell lines [60], as well as histone acetylation [61]. We have previously reported that acetylation of ProT and Smad7 is associated with emphysema progression [19]. In the present study, we further identified that the regulation requires nuclear ProT expression, triggering Smad7 nuclear localization and transcriptional suppression of *SNAI1*, *TWIST1*, and *ZEB1* genes.

## 5 Conclusion

Our study reveals an interesting insight into the biological function and molecular mechanism of ProT in tumor progression. The loss of nuclear ProT expression might serve as an indicator of tumor metastasis. Increased ProT expression is associated with tumor development but is accompanied by TGF‐β signal interruption and EMT suppression. Lung cancer patients who lack nuclear ProT expression might have a higher risk of developing metastasis. Targeting ProT as a therapeutic approach still requires a more in‐depth understanding. Nevertheless, ProT has shown the potential to serve as a biomarker for cancer diagnosis and prognosis and to monitor tumor progression.

## Conflict of interest

The authors declare no conflict of interest.

## Author contributions

LC and B‐HS designed and performed experiments, and analyzed data. C‐TW, L‐HC, Y‐CW, and G‐SS assisted in animal and molecular biology experiments. LC, B‐HS, and A‐LS interpreted data and wrote the manuscript. PW, B‐HS, and C‐LW reviewed and revised the manuscript. J‐MC, Y‐LT, T‐HH, and Y‐TY provided clinical specimens and analyzed data. A‐LS and C‐LW jointly supervised the work. All authors approved the final version of the paper.

## Peer review

The peer review history for this article is available at https://www.webofscience.com/api/gateway/wos/peer‐review/10.1002/1878‐0261.70035.

## Supporting information


**Fig. S1.** Immunohistochemical detection and quantification of total and nuclear ProT in lung tumor specimens.
**Fig. S2.** Nuclear ProT expression in A549/ProT cells and endogenous Snail expression in H1299 cells.
**Fig. S3.** Cytoplasmic ProT promotes tumor metastasis.
**Fig. S4.** Single‐cell RNA sequencing analysis of *PTMA* in lung adenocarcinoma reveals its association with TGF‐β signaling and EMT pathways.
**Fig. S5.** Analysis of gene expression and overall survival of lung cancer patients from the TCGA lung cancer (lung adenocarcinoma LUAD) cohort (*n* = 502).

## Data Availability

The datasets used and/or analyzed during the current study are available from the corresponding author upon reasonable request.
