# Supplementary material for: Aberrant expression of nuclear prothymosin α contributes to epithelial‐mesenchymal transition in lung cancer
Source: Mol Oncol. 2025 Apr 21;19(9):2730–49. doi: 10.1002/1878-0261.70035 (PMC12420368; doi:10.1002/1878-0261.70035)
Supplement: Supplementary file 1 — Fig. S1. Immunohistochemical detection and quantification of total and nuclear ProT in lung tumor specimens. Fig. S2. Nuclear ProT expression in A549/ProT cells and endogenous Snail expression in H1299 cells. Fig. S3. Cytoplasmic ProT promotes tumor metastasis. Fig. S4. Single‐cell RNA sequencing analysis of PTMA in lung adenocarcinoma reveals its association with TGF‐β signaling and EMT pathways. Fig. S5. Analysis of gene expression and overall survival of lung cancer patients from the TCGA lung cancer (lung adenocarcinoma LUAD) cohort (n = 502). [file MOL2-19-2730-s001.docx]

**Supporting information for**

**Aberrant expression of nuclear prothymosin α contributes to epithelial-mesenchymal transition in lung cancer**

Liyun Chen *et al.*

**Supplementary Figures**


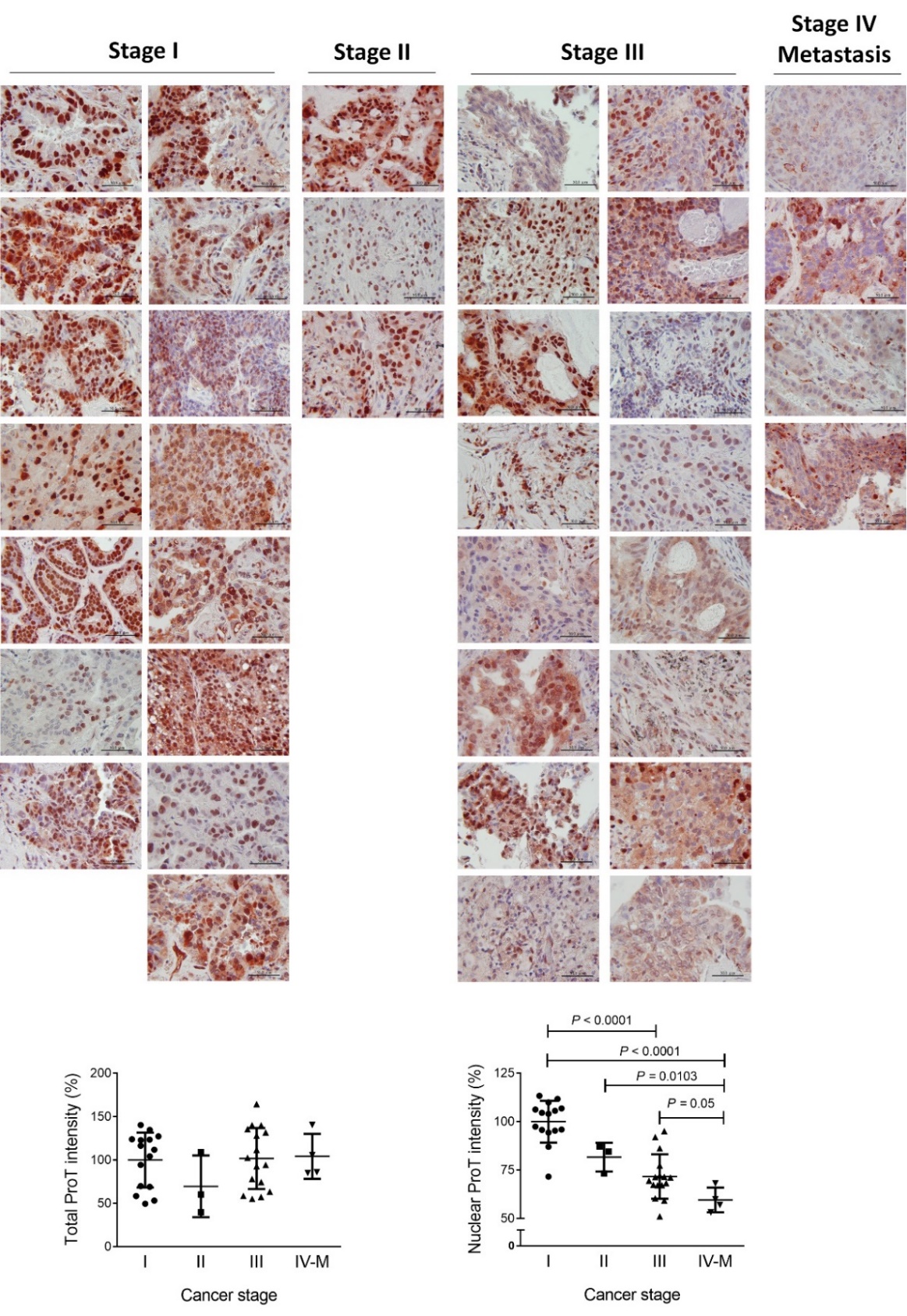


**Fig. S1.** **Immunohistochemical detection and quantification of total and nuclear ProT in lung tumor specimens.** Values shown are levels of immunointensity in individual specimens, with mean levels in stage I tumors arbitrarily set to 100, in three randomly selected fields in each section. Horizontal bars represent the mean ± SEM (n = 15 for stage I; n = 3 for stage II; n = 16 for stage III; n = 4 for stage IV-M). Scale bars: 50 μm. SEM, standard error of the mean.


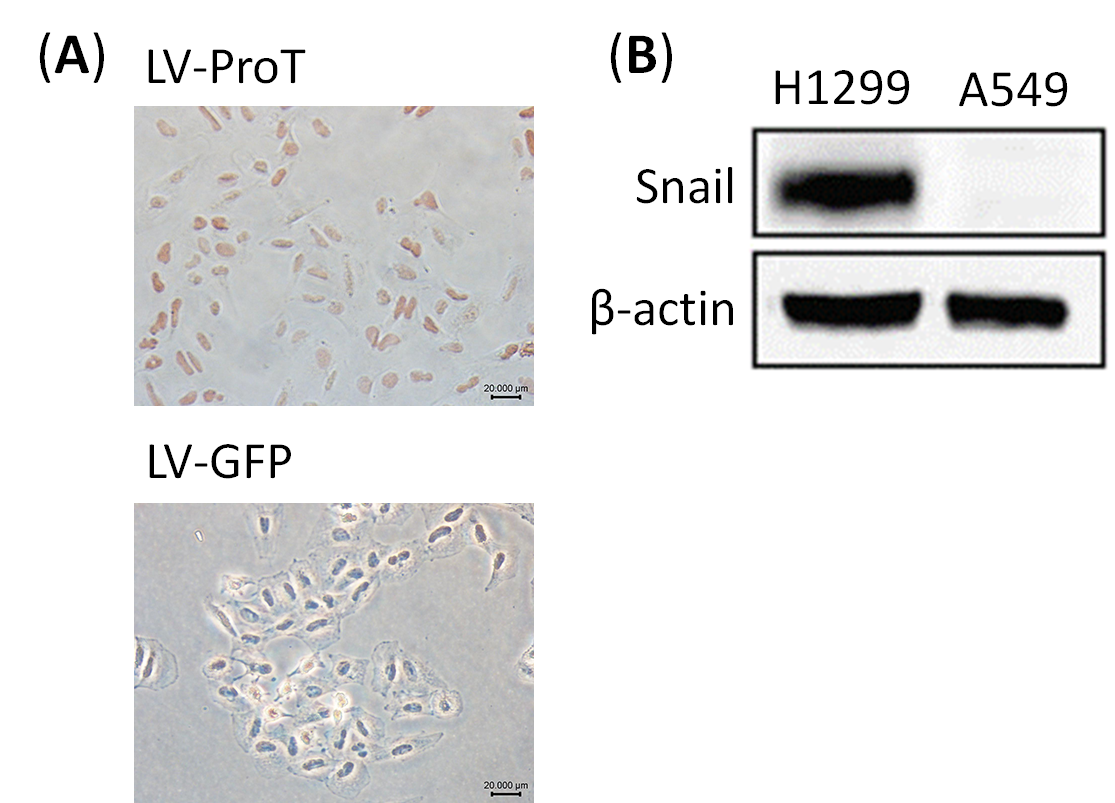


**Fig. S2.** **Nuclear ProT expression in A549/ProT cells and endogenous Snail expression in H1299 cells.** (A) Nuclear ProT expression was detected by immunostaining in A549/ProT and A549/GFP cells. Scale bars shown on images correspond to 20 μm. (B) Expression of endogenous Snail in H1299 and A549 human lung cancer cell lines was determined by immunoblotting.


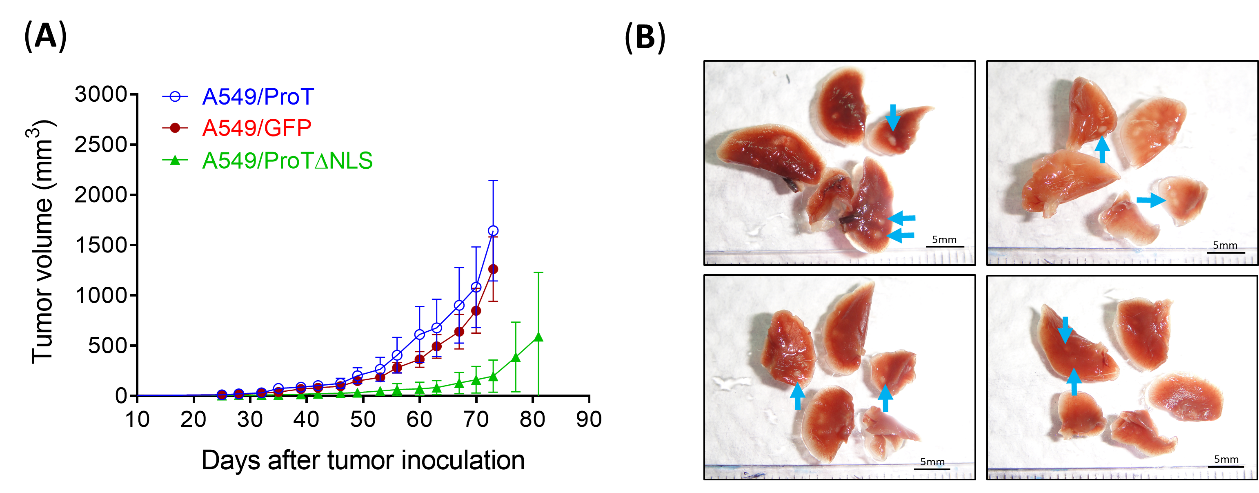


**Fig. S3. Cytoplasmic ProT promotes tumor metastasis.** Groups of 8 NOD/SCID mice were subcutaneously inoculated with A549/GFP, A549/ProT, or A549/ProTΔNLS lacking the NLS (4 ×10^6^) at day 0, and tumor volumes were measured every 3-4 days. Mice were sacrificed at day 73 (or day 81 for the A549/ProTΔNLS group). (A) Tumor volumes of mice bearing different A549 tumors. Note that A549/ProTΔNLS tumors grew at a slower rate than A549/ProT or A549/GFP tumors. Error bars indicated mean ± SEM. (B) Gross appearance of the lungs from A549/ProTΔNLS tumor-bearing mice suggests tumor metastasis. Arrowheads indicate tumor nodules. Note that 4 out of 8 mice bearing A549/ProTΔNLS tumors developed lung metastasis. Scale bars shown on images correspond to 5 mm. SEM, standard error of the mean.


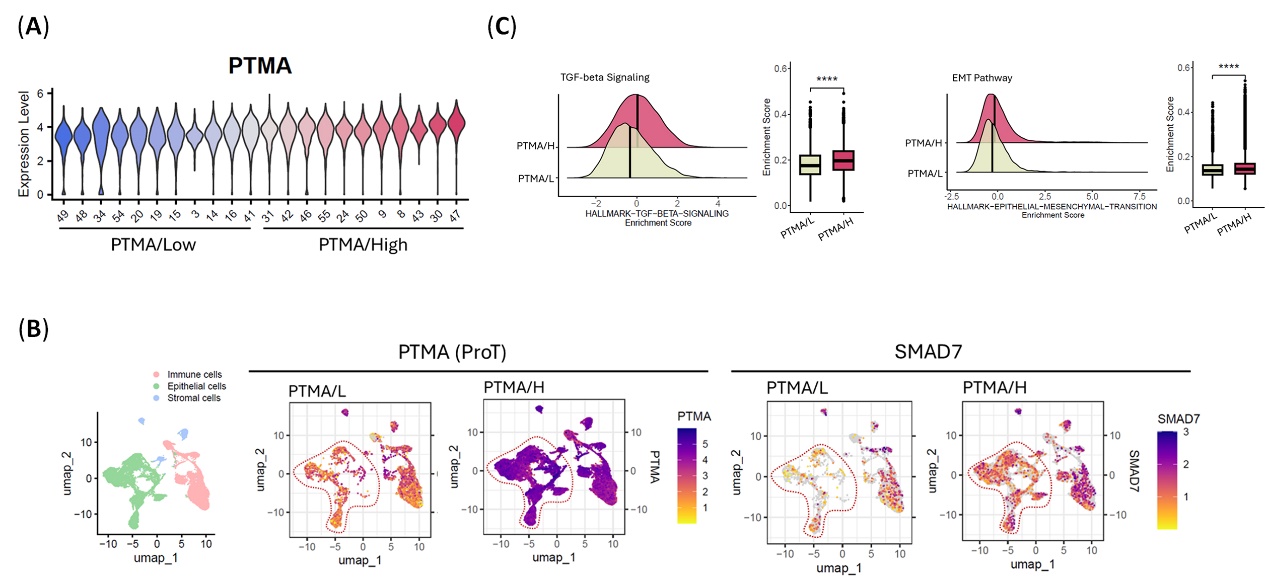


**Fig. S4. Single-cell RNA sequencing analysis of *PTMA* in lung adenocarcinoma reveals its association with TGF-β signaling and EMT pathways.** (A) Violin plot shows the distribution of *PTMA* transcript levels from a single-cell RNA sequencing dataset of 22 patients with lung adenocarcinoma (GSE148071). Patients were divided into *PTMA*-High (*PTMA*/H, n = 11) and *PTMA*-Low (*PTMA*/L, n = 11) groups based on *PTMA* expression levels in epithelial cells. (B) U-MAP shows the expression of *PTMA* and *SMAD7* in lung adenocarcinoma. Cancer epithelial cells with high *PTMA* expression showed a positive correlation with higher *SMAD7* expression compared to the *PTMA*-L group (red dotted line highlighted the epithelial cells). (C) Gene set enrichment analysis comparing enriched signaling in *PTMA*-H and *PTMA*-L groups shows that *PTMA*-H had significantly higher enrichment scores for both TGF-β signaling and EMT pathways, suggesting that high *PTMA* expression is associated with enhanced metastatic potential in lung adenocarcinoma.


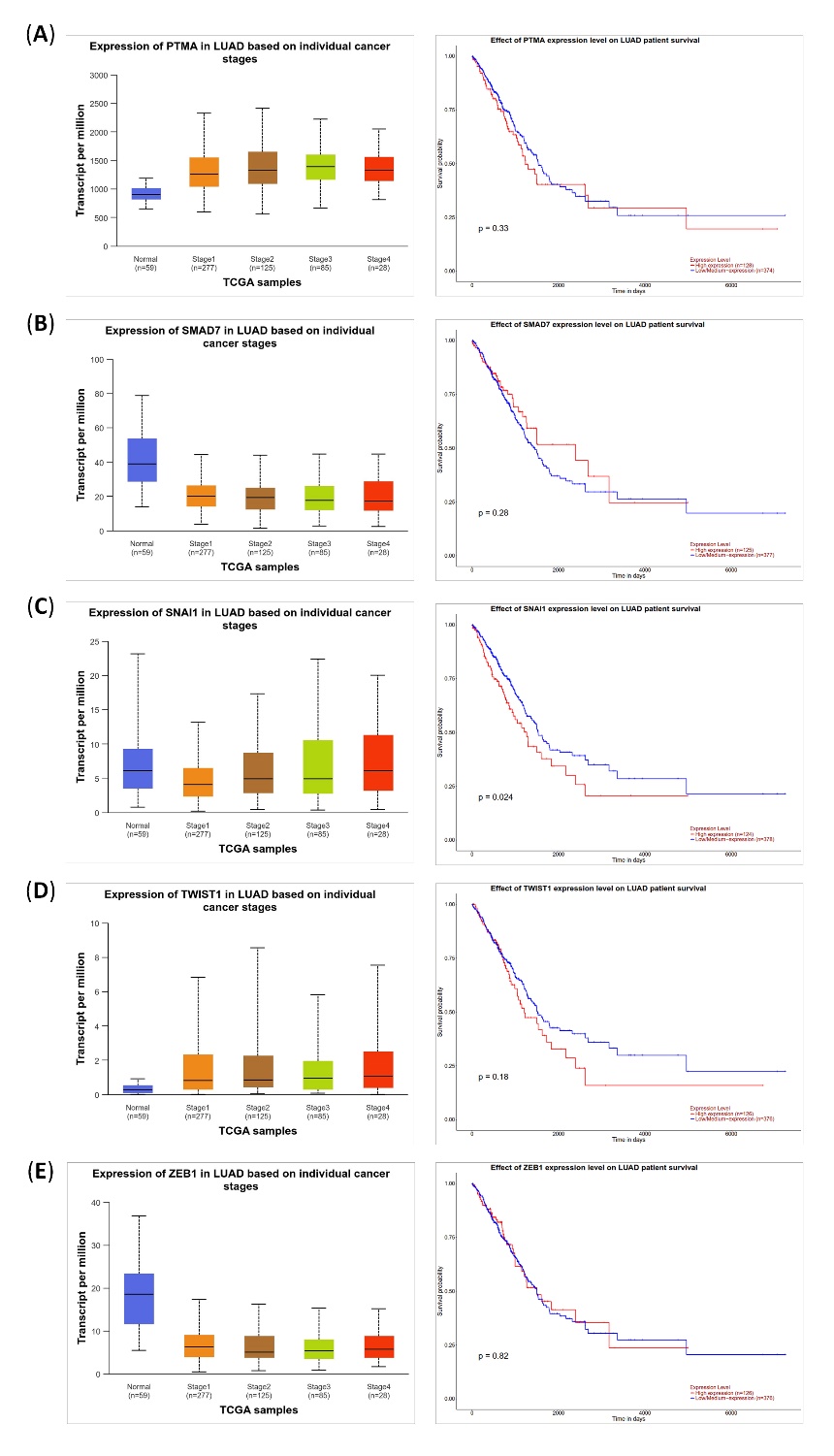


**Fig. S5. Analysis of gene expression and overall survival of lung cancer patients from the TCGA lung cancer (lung adenocarcinoma LUAD) cohort (n = 502).** (A-E, *left*) Gene expression levels of *PTMA* (A), *SMAD7* (B), *SNAI1* (C), *TWIST1* (D), and *ZEB1* (E) in different cancer stages (I-IV). Error bars indicated mean ± SEM. (A-E, *right*) Kaplan-Meier curves of overall survival of lung cancer patients. The overall survival was grouped according to the expression levels of *PTMA* (A), *SMAD7* (B), *SNAI1* (C), *TWIST1* (D), and *ZEB1* (E). SEM, standard error of the mean. TCGA, The Cancer Genome Atlas.
